# CD147: a critical factor in normal and pathological pregnancies

**DOI:** 10.3389/fmed.2026.1757302

**Published:** 2026-03-20

**Authors:** Hangyu Sun, Jingran Gao, Shuqi Yang, Xiaoying Yao

**Affiliations:** Obstetrics & Gynecology Hospital of Fudan University, Shanghai Key Lab of Reproduction and Development, Shanghai Key Lab of Female Reproductive Endocrine Related Diseases, Shanghai, China

**Keywords:** CD147, maternal-fetal interface, placenta, pregnancy, trophoblast

## Abstract

CD147 (Basigin/EMMPRIN) is a widely expressed trans-membrane glycoprotein. While its role in tumorigenesis is well-established, its functions in the process of pregnancy remain to be systematically elucidated. This review summarizes the expression patterns and functional mechanisms of CD147 in both normal and pathological pregnancies. During normal pregnancy, CD147 plays a critical role in embryo implantation, placental development, and pregnancy maintenance by regulating the expression of matrix metalloproteinases (MMPs), promoting angiogenesis, participating in the construction of the maternal-fetal immune micro-environment, and modulating placental energy metabolism. In pathological pregnancies, aberrant CD147 expression is closely associated with various obstetric complications: its down-regulation is linked to pre-eclampsia, recurrent implantation failure, and fetal growth restriction, whereas its up-regulation may promote invasive behaviors in gestational choriocarcinoma and placenta accreta spectrum. This review emphasizes the “dual regulatory” characteristic of CD147 in pregnancy, highlighting its potential as a biomarker and therapeutic target, and proposes directions for future research.

## 1 Introduction

CD147, also known as Basigin or Extracellular Matrix Metalloproteinase Inducer (EMMPRIN), is a multifunctional trans-membrane glycoprotein belonging to the immunoglobulin (Ig) super-family, garnering significant attention for its central role in regulating cell-cell communication and cell-microenvironment interactions (1, 2).

### 1.1 Structure of CD147

Its encoding gene is located on chromosome 19p13.3, and the molecular weight of the encoded protein varies between 43 and 66 kDa depending on its glycosylation status (1, 3). The most common isoform is the widely expressed Basigin-2, whose structure comprises a short intracellular domain, a trans-membrane region, and a characteristic extracellular region containing two Ig-like domains (4, 5). Another major isoform, Basigin-1, contains three Ig-like domains and is expressed specifically mainly in the retina (6). The trans-membrane and intracellular domains of CD147 are highly conserved throughout evolution, suggesting these regions are crucial for its function (7–9).

Furthermore, there are three glycosylation sites at the N-terminus of its extracellular segment. Varying degrees of glycosylation not only contribute to its molecular weight diversity but also directly regulate its functional activity (10, 11). For instance, the highly glycosylated form of CD147 (HG-CD147, 40–65 kDa) has been demonstrated to be the key active form that promotes the membrane localization and function of monocarboxylate transporters (MCT1/4), thereby regulating cellular metabolism (3, 10, 12).

### 1.2 Function of CD147

CD147 is a well-established promoter of tumor progression, and its key mechanisms provide a valuable framework for understanding its roles in pregnancy. Its most characterized function is inducing matrix metalloproteinases (MMPs), thereby facilitating extracellular matrix remodeling and cell invasion-a process highly relevant to trophoblast biology (13–16). CD147 also drives epithelial-mesenchymal transition (EMT) and promotes angiogenesis, largely through up-regulating vascular endothelial growth factor (VEGF) (17–19). Moreover, as an essential chaperone for monocarboxylate transporters (MCT1/4), CD147 sustains glycolytic metabolism (the Warburg effect) and maintains intracellular pH homeostasis, which is critical for rapid cell proliferation (14, 20, 21). Beyond these cell-autonomous effects, CD147 profoundly shapes the tumor microenvironment, particularly by modulating immune responses. It facilitates immune evasion through interactions with ligands such as Cyclophilin A (CyPA), influencing T-cell function and cytokine profiles (22–25). These oncogenic mechanisms-MMP induction, angiogenesis, metabolic reprogramming, and immune modulation-are strikingly paralleled in placental development, as discussed in the following sections.

### 1.3 Mechanism of action of CD147

**FIGURE 1:**
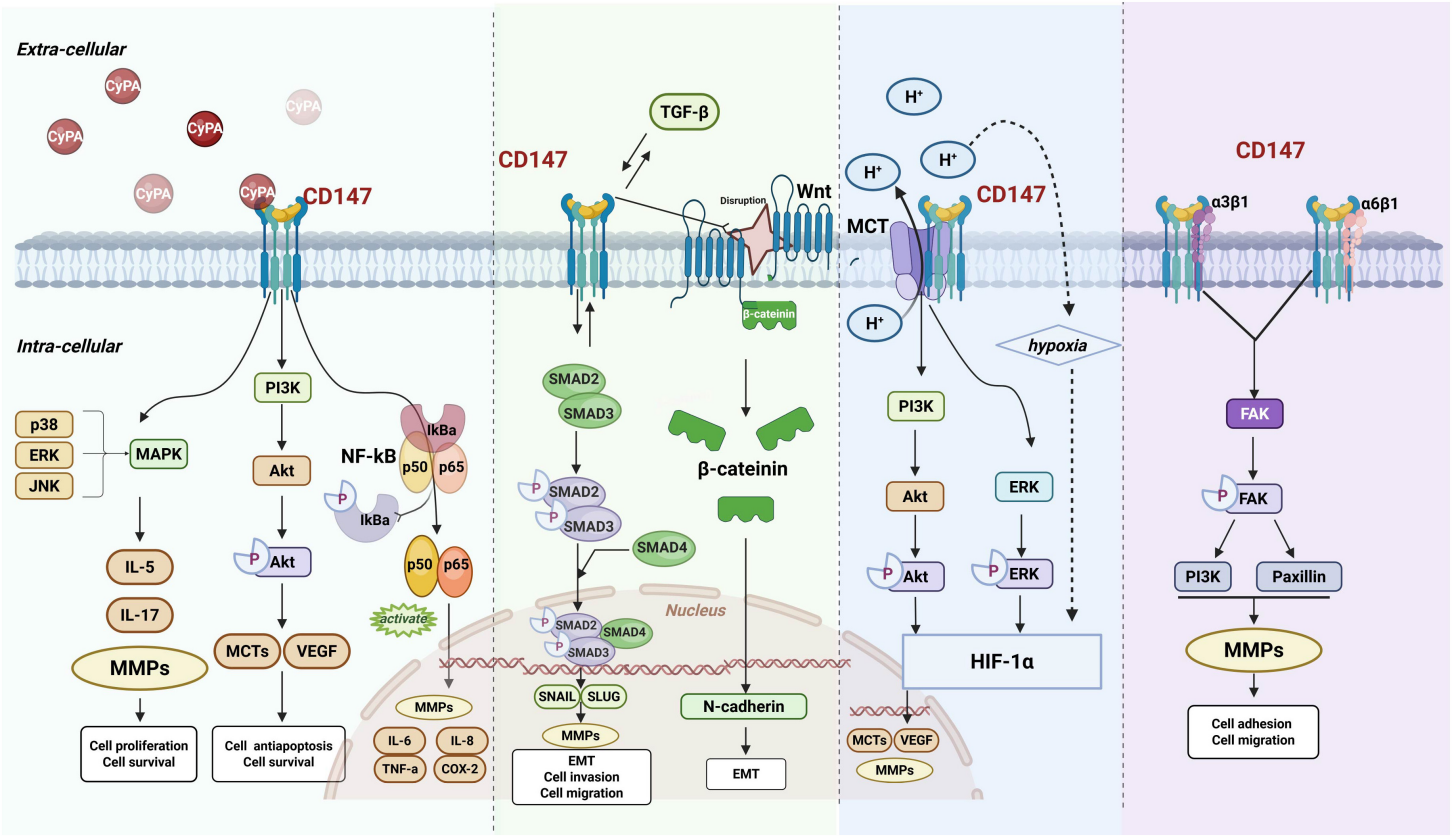
The biochemical mechanisms of CD147. CyPA, Cyclophilin A; ERK, extracellular regulated protein kinases; JNK, c-Jun N-terminal kinase; MAPK, mitogen-activated protein kinase; IL-5, Interleukin-5; IL-17, Interleukin-17; MMPs, matrix metalloproteinases; PI3K, Phosohatidyqinositol-3 kinase; Akt, protein kinase B; MCTs, monocarboxylate transporters; VEGF, vascular endothelial growth factor; NF-κB, nuclear factor kappa-B; IL-6, Interleukin-6; IL-8, Interleukin-8; TNF-α, tumor necrosis factor α; COX-2, Cyclooxygenase-2; TGF-β, transforming growth factor-β; EMT, epithelial-mesenchymal transition; HIF-1α, hypoxia-inducible factor 1α; FAK, Focal Adhesion Kinase.

CD147 functions as a multifunctional signaling hub that integrates diverse pathways to regulate cellular behavior (Figure 1). Its core mechanism begins with binding to the extracellular ligand Cyclophilin A (CyPA), an interaction that directly activates downstream key signaling pathways such as ERK/MAPK (26, 27), PI3K/Akt (28, 29), and NF-κB (30, 31). This activation drives cell proliferation, survival, and the expression of inflammatory cytokines and MMPs, while also promoting chemotherapy resistance (31, 32). In inducing epithelial-mesenchymal transition (EMT), CD147 forms a positive feedback loop with TGF-β signaling and activates the Wnt/β-catenin pathway (26, 27). By upregulating transcription factors like Snail and Slug and disrupting the E-cadherin-β-catenin complex, it collectively enhances cell migration and invasion capabilities (33, 34). Simultaneously, CD147 acts as an essential molecular chaperone for monocarboxylate transporters (MCT1/4), ensuring their membrane localization and function, and mediating lactate efflux to sustain glycolytic metabolism (1, 9). This process forms a positive feedback loop with the hypoxia-inducible factor (HIF-1α), further upregulating VEGF and MMPs, and synergistically promoting angiogenesis and tumor progression (29). Furthermore, CD147 interacts with integrins (such as α3β1 and α6β1) to activate Focal Adhesion Kinase (FAK) and its downstream signals, precisely regulating cell adhesion and motility (5). Beyond these core pathways, CD147’s functions extend to forming complexes with CD98hc to regulate metabolism, activating STAT3 signaling to maintain stem cell properties (33), mediating DNA repair via the ATM/ATR/p53 pathway (29), and participating in the hyaluronic acid-CD44 axis-driven drug resistance (34), collectively forming its complex functional network.

## 2 The role of CD147 in normal pregnancy

### 2.1 Expression and localization of CD147 in gestational tissues

CD147 (Basigin/EMMPRIN) exhibits a highly spatiotemporally specific expression pattern in various cells at the maternal-fetal interface. Its expression level is hormonally regulated and closely associated with the progression of pregnancy (Table 1).

**TABLE 1:** Expression patterns of CD147 in human gestational tissues.

Tissue/cell type	Expression pattern	Gestational timing	Proposed function	Key reference
Endometrium (glandular epithelium)	High in proliferative phase; decreases in secretory phase	Menstrual cycle	Epithelial remodeling, receptivity	(35, 36)
Endometrial stroma/decidua	Increases in secretory phase; peaks during implantation	Peri-implantation	Stromal decidualization, immune cell recruitment	(39–41)
Cytotrophoblasts (CTBs)	High in first-trimester; decreases later	Early pregnancy	Proliferation, initial invasion	(43, 44)
Syncytiotrophoblasts (STBs)	High expression	Throughout pregnancy	Barrier function, secretion	(44)
Extravillous trophoblasts (EVTs)	Sustained in basal decidua	Mid to late pregnancy	Invasion, spiral artery remodeling	(43, 47)
Placental endothelial cells	Moderate	Mid to late pregnancy	Angiogenesis, vascular stability	(46)

#### 2.1.1 Endometrium and decidua

In the human endometrium, CD147 expression shows a clear menstrual cycle dependence. During the proliferative phase, it is primarily expressed in glandular epithelial cells. In the secretory phase, epithelial expression weakens, while expression in stromal cells gradually intensifies and expands from the luminal toward the basal layer (35, 36). This expression pattern is also confirmed in baboon models, suggesting coordinated regulation by estrogen and progesterone (37). The expression level of CD147 in the endometrium of patients with recurrent implantation failure (RIF) is significantly lower than that in normally fertile women (38), highlighting its importance for endometrial receptivity. In mouse models, CD147 expression demonstrates distinct spatiotemporal changes: it is mainly expressed in luminal and glandular epithelium on gestation days 1–2, shifting to the stromal layer by day 4 (implantation period), and shows strong positivity in stromal cells surrounding the implanting embryo (cells about to decidualize). As these cells differentiate into mature decidual cells, its expression is rapidly down-regulated and confined to undifferentiated stromal cells (39–41). *In vitro* experiments confirm that CD147 is crucial for the proliferation and decidualization process of human endometrial stromal cells (HESCs) (42). CD147 expression changes dynamically throughout pregnancy. In the endometrium, its expression is upregulated during the secretory/peri-implantation period to prepare for embryo implantation and downregulated after decidualization is complete.

#### 2.1.2 Placental trophoblast cells

CD147 is abundantly expressed in various trophoblast cell types of the human placenta. In first-trimester villi, it is highly expressed in cytotrophoblasts (CTBs), syncytiotrophoblasts (STBs), and columnar cytotrophoblasts; by the mid-to-late trimester, its expression stabilizes in extravillous trophoblasts (EVTs) of the basal decidua, suggesting its association with pregnancy maintenance (43). Studies confirm abundant expression of CD147 in human primary trophoblasts, trophoblast cell lines (e.g., BeWo), and in CTBs and STBs of first-trimester placental tissue (44). The protein and mRNA expression levels of CD147 in placental tissue from preeclamptic (PE) patients are significantly lower than those in the normal pregnancy group (45). In the mouse placental labyrinth, CD147 mRNA is widely and highly expressed, and its protein co-localizes with the monocarboxylate transporters MCT1 and MCT4, collectively constituting the placental barrier for substance transport (46).

In the placental trophoblast, its expression is strongest in the first trimester (e.g., 6 weeks gestation) and gradually decreases in the villi as pregnancy progresses, but remains stable in the basal decidua, suggesting a shift in its function from promoting invasion early on to maintaining homeostasis and facilitating substance transport in later stages (43).

### 2.2 Physiological functions of CD147 in pregnancy

#### 2.2.1 Trophoblast invasion

Substantial evidence indicates that CD147 regulates the invasive behavior of trophoblasts by inducing the expression of matrix metalloproteinases (MMPs), a mechanism highly analogous to that in tumor cells. In mouse uterine stromal cells, recombinant CD147 protein can significantly induce the mRNA expression and protein secretion of MMP-3 and MMP-9 (39). Knocking down CD147 in human endometrial stromal cells leads to a significant decrease in MMP-2 and MMP-3 protein levels (42). Functional studies confirm that inhibiting CD147 with function-blocking antibodies or siRNA in JEG-3 cells and primary EVTs significantly impairs cell invasion ability and MMP2 activity; conversely, using a CD147 agonistic antibody enhances invasion capability (47).

#### 2.2.2 Placental angiogenesis

Based on its well-established role in tumor angiogenesis, CD147 is hypothesized to similarly regulate placental vascular network formation. In oncology, CD147 up-regulates VEGF and synergizes with inflammatory factors to drive new blood vessel growth (36, 48). Given that embryo implantation and placentation are highly angiogenesis-dependent processes, and a positive correlation between VEGF-A and EMMPRIN levels has been observed in blastocyst culture media (49), we propose that CD147 may stimulate VEGF expression in trophoblasts or placental endothelial cells in a paracrine/autocrine manner. However, this hypothesis remains to be directly tested in placental tissues, representing an important direction for future functional studies.

#### 2.2.3 Maternal-fetal immune tolerance

Unlike the mechanism in tumor immune evasion that directly inhibits T-cell function, CD147 at the maternal-fetal interface appears to participate in building the immune microenvironment primarily by actively recruiting rather than inhibiting immune cells. Mouse studies found that recombinant CD147 protein potently induces the production of various inflammatory cytokines (IL-1α, IL-1β) and chemokines (CCL3, CCL20, CXCL2, CXCL5) in uterine stromal cells (39). These factors are key signals for recruiting and activating innate immune cells such as macrophages, neutrophils, and dendritic cells. This suggests that the transient high expression of CD147 at the implantation site may be an important signal initiating the recruitment of immune cells to the uterus. These recruited immune cells (e.g., macrophages) subsequently play vital roles in promoting placental development and establishing immune tolerance. These recruited immune cells, particularly macrophages and dendritic cells, subsequently contribute to the establishment of fetal-specific immune tolerance. For instance, decidual macrophages promote an anti-inflammatory micro-environment through the production of IL-10 and indoleamine 2,3-dioxygenase (IDO) (50), while dendritic cells facilitate the induction of regulatory T cells (Tregs), which are essential for maintaining maternal-fetal tolerance (51, 52). Thus, CD147-mediated immune cell recruitment represents an initial step in a cascade that ultimately supports immune tolerance rather than rejection.

#### 2.2.4 Cellular metabolic reprogramming

Glycolysis, as the core process of cellular energy metabolism, plays a fundamental role, especially in malignant tumor cells. Tumor cells still preferentially uptake and utilize glucose through aerobic glycolysis even in the presence of sufficient oxygen, a phenomenon known as the Warburg effect (53). As an essential molecular chaperone for lactate transporters (MCT1 and MCT4) (54), CD147 is directly involved in regulating placental energy metabolism, closely resembling the “Warburg effect” in tumor cells. Studies confirm that CD147 co-localizes with MCT1 and MCT4 on the trophoblast cell membrane in the mouse placental labyrinth, ensuring these transporters are correctly anchored to the membrane to perform their function of transporting monocarboxylates like lactate and ketone bodies across the membrane, which is crucial for maintaining intracellular energy metabolism and pH homeostasis (44, 46). It is worth noting that although the knockout of CD147 does not affect the mRNA expression of MCT1/4, it seriously affects its transport from the endoplasmic reticulum to the plasma membrane, thereby reducing lactate output (55). Given that the late-gestation placenta is a high-energy-consumption organ exhibiting active glycolysis, the CD147-MCTs complex is a key hub supporting placental energy metabolic reprogramming and rapid fetal growth (36, 46).

## 3 The role of CD147 in pathological pregnancies

### 3.1 Preeclampsia

Extensive clinical evidence and functional experiments indicate that reduced CD147 expression or function is a significant pathogenic factor in preeclampsia (PE). This conclusion is supported by multi-level validation from clinical samples to animal models and *in vitro* experiments. Compared to normal pregnancy, the protein and mRNA expression levels of CD147 in placental tissue from PE and eclamptic patients are significantly decreased (45). More importantly, as early as the first trimester, the levels of soluble CD147 in the placental tissue and serum of women who later develop PE (especially early-onset) are already significantly reduced (47), suggesting CD147 could serve as an early predictive biomarker for PE. Furthermore, a CD147 gene polymorphism (rs424243T/G) has been found to be significantly associated with PE risk, with pregnant women carrying the G variant genotype having an increased risk of the disease (56), supporting the fundamental role of CD147 in PE pathogenesis from a genetic perspective.

This may be directly related to impaired trophoblast invasion capacity caused by reduced CD147 function. *In vitro* experiments show that inhibiting CD147 expression or function significantly weakens trophoblast invasion ability, a mechanism associated with the inhibition of MMP-2, MMP-9, and urokinase-type plasminogen activator enzymatic activity (44). Animal models directly confirm that specific knockdown of CD147 in mouse trophoblasts using nanoparticle technology is sufficient to successfully induce typical PE-like symptoms, including hypertension, proteinuria, placental hypoperfusion, and fetal growth restriction (47). This model further revealed that CD147 deficiency disrupts normal placental development, leading to a reduced number of invasive trophoblast cells and inhibited differentiation of extra villous trophoblasts (57). Additionally, CD147 deficiency is associated with impaired angiogenesis. In a mouse model with uterus-specific CD147 knockout, reduced expression of CD31 (a vascular endothelial marker) was observed, suggesting diminished angiogenesis (40). This provides a potential explanation for the inadequate spiral artery remodeling and placental ischemia observed in PE.

The association between reduced CD147 and PE requires nuanced interpretation. The link appears more pronounced in early-onset than late-onset PE, suggesting pathogenic heterogeneity. Study variations in sample type, timing, and methodology also contribute to inconsistent findings. Furthermore, compensatory upregulation of other factors may modulate the phenotypic severity of PE despite CD147 reduction. Thus, future studies should stratify by PE subtype and standardize detection methods to clarify CD147’s precise diagnostic utility.

### 3.2 Spontaneous abortion/embryo implantation failure

Abnormal CD147 expression is closely associated with embryo implantation failure and early pregnancy loss. The expression of CD147 (including in glandular epithelium, stroma, and vascular endothelial cells) in the endometrium of RIF patients is significantly lower than in women with normal fertility (38). Insufficient CD147 expression may lead to abnormal endometrial remodeling, impair endometrial receptivity, and thus affect embryo implantation, which is considered a potential mechanism for RIF (38). Animal models strongly demonstrate the indispensability of CD147 for successful implantation. Most homozygous embryos with global CD147 gene knockout die around the peri-implantation period (58). Notably, even wild-type embryos, when transferred into CD147-deficient mother mouse uteri, exhibit very low delivery efficiency (59), proving that maternal uterine expression of CD147 is also crucial for supporting embryo implantation. Therefore, functional defects in CD147 in either the mother or the embryo itself can lead to spontaneous abortion.

### 3.3 Fetal growth restriction

Decreased CD147 expression may contribute to the pathogenesis of fetal growth restriction by affecting placental function and nutrient transport. As mentioned earlier, CD147-deficient PE mouse models are consistently accompanied by fetal growth restriction (47). Beyond invasion and angiogenesis abnormalities, CD147’s function as a chaperone for MCT1/MCT4 is vital for placental substance transport. CD147 deficiency may lead to disrupted membrane localization of MCT1, thereby impairing the transport of key energy substrates like lactate, which could ultimately compromise fetal nutrient supply and lead to growth restriction (40, 42).

### 3.4 Gestational trophoblastic disease

In gestational trophoblastic diseases, the expression pattern of CD147 is highly similar to that in malignant tumors, and its high expression is closely related to the aggressiveness of the disease. CD147 expression is significantly enhanced in choriocarcinoma, the most malignant form, with its level markedly higher than in normal placenta, partial hydatidiform mole, and complete hydatidiform mole (60, 61). This aligns with the highly invasive and metastatic characteristics of choriocarcinoma. In choriocarcinoma, CD147 promotes extracellular matrix degradation by inducing the expression of MMPs (e.g., MMP-1, MMP-2, MMP-14), thereby enhancing tumor cell invasion and metastatic capacity (61). Furthermore, studies confirm that CD147 is an important surface marker for circulating tumor cells in choriocarcinoma. Anti-CD147 antibodies can be used to capture these circulating cells, and their count significantly correlates with higher FIGO stage and chemotherapy resistance (62). This makes CD147 a highly promising biomarker and therapeutic target for gestational trophoblastic diseases, especially choriocarcinoma, in disease monitoring, risk stratification, and treatment efficacy evaluation (62).

### 3.5 Placenta accreta spectrum disorders

In contrast to the insufficient trophoblast invasion associated with low CD147 expression in PE and RIF, we hypothesize that CD147 may be abnormally upregulated in placenta accreta spectrum disorders, thereby potentially driving pathological over-invasion. This hypothesis is grounded in two key indirect observations from the literature. First, MMPs such as MMP-2 and MMP-9 are significantly upregulated in placenta accreta tissues (63, 64). Given that CD147 is a well-established key inducer of MMPs–a mechanism robustly demonstrated in highly invasive, CD147-expressing malignancies like choriocarcinoma (60). It is plausible that elevated CD147could contribute to the MMP overexpression seen in placenta accreta. Second, placenta accreta lesions are characterized by high VEGF expression and uncontrolled angiogenesis (65, 66), which aligns with CD147’s known function in tumors to activate the VEGF pathway and synergize with inflammatory factors to promote angiogenesis (67).

**FIGURE 2:**
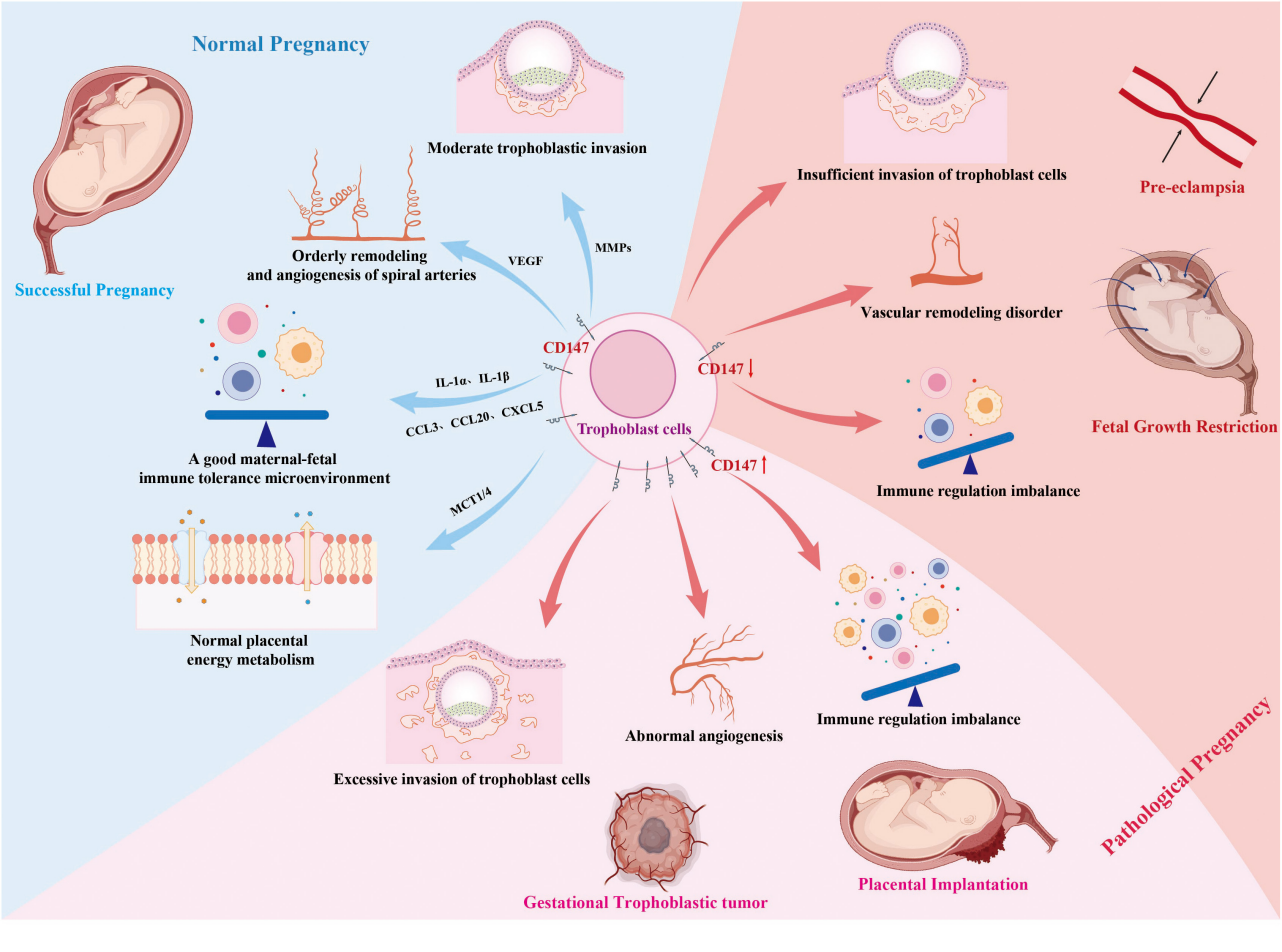
The mechanism of CD147 in physiological and pathological pregnancy.

In summary, the expression level and functional status of CD147 play a central role in the pathogenesis of various pathological pregnancies (Figure 2), exhibiting a remarkable “dual regulatory” pattern. On one hand, its down-regulation or functional impairment is a key factor leading to insufficient trophoblast invasion, closely related to the pathological basis of early-onset (placental) pre-eclampsia, recurrent implantation failure fetal growth restriction; On the other hand, its upregulation or hyper-function may drive invasive behaviors, as seen in gestational trophoblastic tumors like choriocarcinoma, as we reasonably speculate, in placenta accreta spectrum disorders. These opposing roles in vastly different pathological states not only highlight the importance of precise regulation of CD147 in maintaining pregnancy homeostasis but also suggest its potential as a candidate biomarker and future therapeutic target (Table 2). However, current evidence remains preclinical; standardized assays, validated thresholds, and safety assessments for pregnancy-directed interventions are still lacking. Further research is needed to translate these mechanistic insights into precise diagnostic and therapeutic strategies for different pathological pregnancies.

**TABLE 2:** CD147 in pathological pregnancies and potential mechanisms.

Condition	CD147 expression change	Proposed mechanisms	References
Pre-eclampsia (early)	↓ (protein and mRNA in placenta)	Impaired trophoblast invasion, defective spiral artery remodeling, disrupted MCT-mediated transport	(45, 47, 57)
Recurrent implantation failure (RIF)	↓ in endometrium	Abnormal endometrial remodeling, impaired stromal decidualization	(38, 42)
Fetal growth restriction (FGR)	↓ (often with PE)	Compromised nutrient transport via MCT1/4 dysregulation	(40, 47)
Gestational choriocarcinoma	↑↑ (vs. normal placenta)	Highly-expressed MMPs, promotion of angiogenesis, metabolic reprogramming.	(60–62)
Placenta accreta spectrum	Hypothesized ↑ (based on MMPs/VEGF upregulation)	Putative MMP/VEGF over-activation, similar to choriocarcinoma	(63–67)

↑ CD147 expression increases; ↓ CD147 expression decreases.

## 4 Discussion

Despite the compelling parallels between CD147 in tumor and trophoblast biology, several limitations and knowledge gaps must be acknowledged. First, many mechanistic insights derive from cancer cell lines or animal models, which may not fully recapitulate the unique spatiotemporal regulation of CD147 in human placenta. For instance, CD147 expression varies significantly across gestational ages and placental regions, suggesting context-specific functions that are not yet fully understood (43, 46). Second, direct extrapolation of tumor-derived mechanisms to pregnancy requires caution, given the fundamentally different physiological endpoints (controlled invasion vs. malignant metastasis). Third, clinical studies on CD147 in pregnancy complications often show heterogeneity – e.g., differences between early- and late-onset preeclampsia, or variability in assay methods – which complicates the establishment of consistent diagnostic thresholds (47, 56). Future research should prioritize human primary tissue models, single-cell omics approaches, and well-phenotyped longitudinal cohorts to validate CD147 as a reliable biomarker and safe therapeutic target.

This review systematically elucidates the dual regulatory role of CD147 in the pregnancy process, a characteristic that presents significant opportunities for clinical translation while also posing challenges that require careful consideration. From a clinical value perspective, CD147 demonstrates considerable potential as a biomarker for pregnancy-related disorders. Research suggests that dynamic changes in soluble CD147 levels in maternal peripheral blood could serve as an important indicator for monitoring pregnancy status – its decreasing trend in early PE offers a new approach for prediction, while its upregulated expression in placenta accreta spectrum and gestational trophoblastic tumors may provide new references for the diagnosis and prognosis assessment of these conditions. Based on its expression characteristics in pathological pregnancies, CD147 also emerges as a potential therapeutic intervention target. Particularly in diseases involving excessive trophoblast invasion, targeted inhibition of CD147 function might offer new therapeutic avenues for controlling disease progression. However, it is imperative to recognize the critical role of CD147 in maintaining normal pregnancy, necessitating a careful balance between the therapeutic benefits of any intervention and its potential impact on physiological pregnancy processes when developing related strategies.

Given the substantial potential and complexity of clinical translation, research in this field needs continuous deepening at multiple levels. First, more precise experimental models, such as trophoblast-specific genetically modified animal models (68, 69) and placental organoid culture systems (70), are required to verify the specific mechanisms of CD147 in different pathological states. Second, the complex regulatory network of CD147 within the maternal-fetal interface micro-environment should be deeply dissected to clarify its stage-specific and cell-type-specific functions, which forms the molecular basis for precise intervention. Breakthroughs in these basic research areas will directly propel clinical translation, including the development of placenta-targeted delivery systems to maximize therapeutic effects while protecting its normal physiological functions. Despite numerous challenges, in-depth research on the role of CD147 is bound to open new avenues for understanding pregnancy physiology and preventing/treating related diseases.

Despite its therapeutic promise, targeting CD147 during pregnancy raises significant safety concerns. CD147 is ubiquitously expressed and essential for neural, immune, and metabolic functions; systemic inhibition risks off-target toxicity. Moreover, as highlighted throughout this review, CD147 is critical for normal placental development, and any intervention must preserve physiological trophoblast function. These considerations underscore the need for precision delivery strategies, such as placenta-targeted nanoparticles or antibody-ligand conjugates, to confine therapeutic effects while minimizing systemic exposure and fetal risk. Developing such approaches is essential for translating CD147-targeted therapies into obstetric medicine.
